# New macronarian from the Middle Jurassic of Chongqing, China: phylogenetic and biogeographic implications for neosauropod dinosaur evolution

**DOI:** 10.1098/rsos.220794

**Published:** 2022-11-02

**Authors:** Hui Dai, Chao Tan, Can Xiong, Qingyu Ma, Ning Li, Haidong Yu, Zhaoying Wei, Ping Wang, Jian Yi, Guangbiao Wei, Hailu You, Xinxin Ren

**Affiliations:** ^1^ No. 208 Hydrogeological and Engineering Geological Team, Chongqing Bureau of Geological and Mineral Resource Exploration and Development, Chongqing, People's Republic of China; ^2^ Chongqing Key Laboratory of Paleontology and Paleoenvironment Co-evolution (Sichuan-Chongqing Joint Construction), Chongqing, People's Republic of China; ^3^ Chongqing Institute of Geological Survey, Chongqing, People's Republic of China; ^4^ Key Laboratory of Vertebrate Evolution and Human Origins, Institute of Vertebrate Paleontology and Paleoanthropology, Chinese Academy of Sciences, Beijing, People's Republic of China; ^5^ CAS Center for Excellence in Life and Paleoenvironment, Beijing, People's Republic of China; ^6^ College of Earth and Planetary Sciences, University of Chinese Academy of Sciences, Beijing, People's Republic of China; ^7^ Key Laboratory of Stratigraphy and Paleontology of the Ministry of Natural Resources, Institute of Geology, Chinese Academy of Geological Sciences, Beijing, People's Republic of China

**Keywords:** Macronaria, Sauropoda, Middle Jurassic, lower Shaximiao formation, phylogeny

## Abstract

Macronaria is a clade of gigantic body-sized sauropod dinosaurs widely distributed from the Late Jurassic to the Late Cretaceous globally. However, its origin, early diversification, and dispersal are still controversial. Here, we report a new macronarian *Yuzhoulong qurenensis* gen. et sp. nov. excavated from the Middle Jurassic (Bathonian) Lower Shaximiao Formation. *Yuzhoulong qurenensis* bears a unique combination of features, such as two accessory fossae that exist on the posterior surface of dorsal diapophyses of anterior dorsal vertebrae. Results of phylogenetic analyses demonstrate it is one of the earliest-diverging macronarians. This new material represents a Middle Jurassic fossil record of macronarian sauropod worldwide and improves the understanding of the early diversity and dispersal of the Neosauropoda. This discovery further supports that sauropods achieved a more rapid and varied morphological diversity and palaeogeographical dispersal in the Middle Jurassic.

## 1. Introduction

The Lower Shaximiao Formation is widely exposed in the Sichuan Basin, China [1]. This unit comprises massive thick purplish-red sandstones and mudstones deposited in a terrestrial (possibly shallow lake) environment [2,3]. The age for this formation is still controversial, traditionally it has been suggested to be Middle Jurassic, based on regional stratigraphic correlations and sedimentology [4–6] and specifically, potentially Bajocian to Bathonian based on some invertebrate remains [7–12], but Bathonian-Callovian-Oxfordian based on recent detrital zircon geochronological age (e.g. [13–19]). The Lower Shaximiao Formation has yielded the remains of several diverse faunas of terrestrial vertebrates [20–38], including seven sauropod genera (*Shunosaurus*, *Protognathus*, *Omeisaurus*, *Abrosaurus*, *Dashanpusaurus*, *Datousaurus*, *Bashunosaurus*).

**Figure 1.**
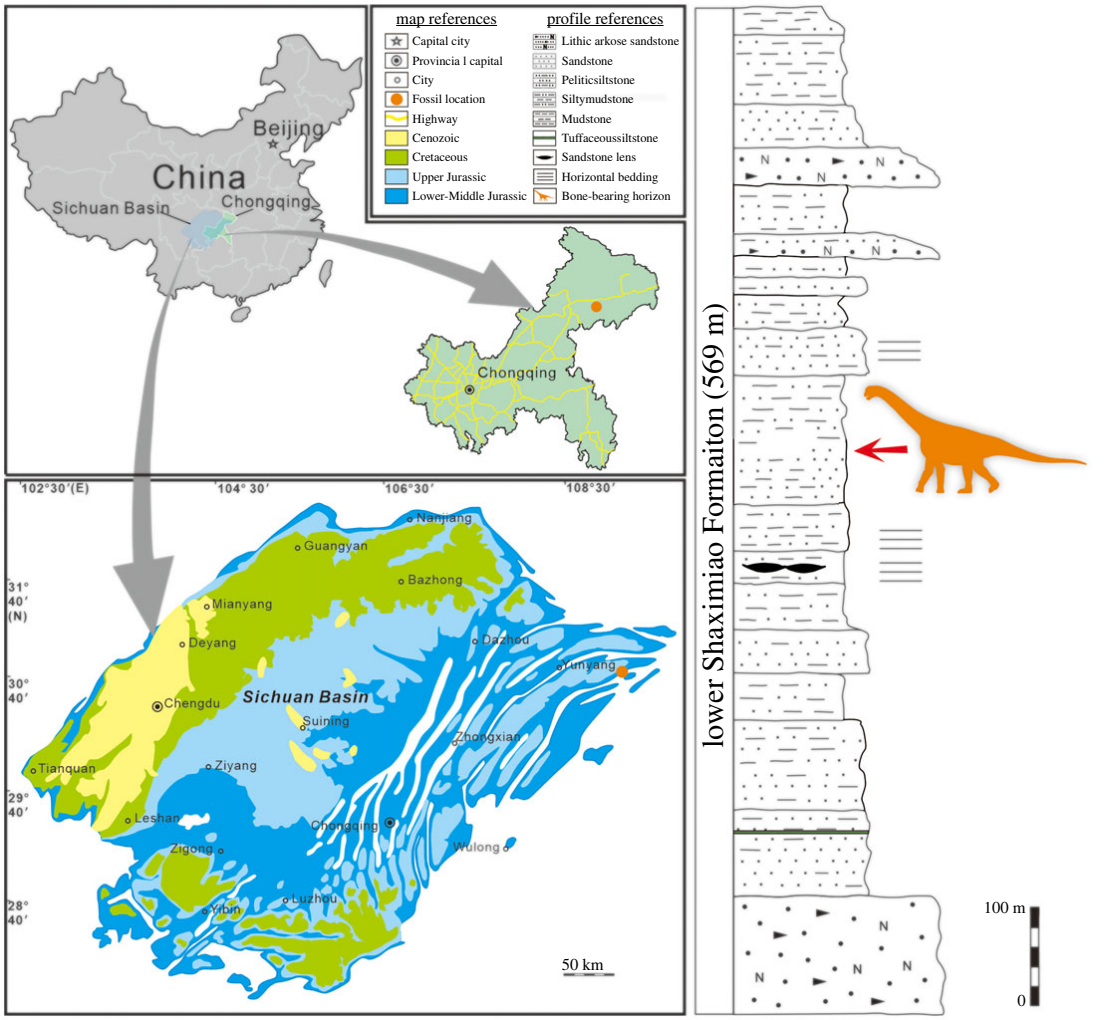
Locality and horizon of *Yuzhoulong qurenensis*.

In 2016, a new Middle Jurassic dinosaur quarry from the Lower Shaximiao Formation was discovered in Pu'an Town, Yunyang, Chongqing Municipality, northeastern Sichuan Basin [38,39]. Here we report a new sauropod specimen, *Yuzhoulong qurenensis* gen. et sp. nov., from this locality (figure 1). It presents a new early branching macronarian morphology and phylogeny. This discovery increases information for a better understanding of the origin, early evolution and paleogeographic distribution of neosauropods.

### 1.1. Anatomical abbreviations

4th, fourth trochanter of femur; ACDL, anterior centrodiapophyseal lamina; acf, anconeal fossa; AF, accessory fossae on the posterior surface of anterior dorsal diapophyses; AL, accessory lamina connecting the SPOL and to the diapophysis of middle dorsal vertebra laterally; ALP, connects the ACDL to PODL in anterior to middle dorsal neural arches; amp, ambiens process; bt, basal tuberae; ca, carotid artery; cd, caudal vertebra; CDF, centrodiapophyseal fossa; ch, chevron; co, coracoid; CPRL, centroprezygapophyseal lamina; d, dorsal; di, diapophysis; dr, dorsal rib; eof, external occipital fenestra for the caudal middle cerebral vein; eo-o, exoccipital–opisthotic complex; fe, femur; fc, fibular condyle; fi, fibula; fo (VII), fenestra ovalis (VII); gl, glenoid; hf (XII), hypoglossal foramen (XII); HIR, the average of the greatest widths of the proximal end, mid-shaft and the distal end of humerus/proximodistal length of the humerus; hpo, hyposphene-hypantrum system; hu, humerus; il, ilium; is, ischium; ls, lateraosphenoid; mc, metacarpal; mf (IX-XI), metotic fenestra (IX-XI); nc, nuchal crest; nsp, neural spine; ocm (III), oculomotor nerve foramen (III); os, orbitosphenoid; pa, parapophysis; PCDL, posterior centrodiapophyseal lamina; pf, lateral pneumatic fossa or foramen; PODL, postzygodiapophyseal lamina; POSDF, postzygapophyseal spinodiapophyseal fossa; pop, paraoccipital process; posta, postacetabular process; poz, postzygapophysis; PPDL, paradiapophyseal lamina; ppr, parasphenoid rostrum; prea, postacetabular process; proot, prootic; PRCDF, prezygapophyseal centrodiapophyseal fossa; prz, prezygapophysis; ptp, pterygoid process; pu, pubis; pua, pubic articulation; puf, pubic foramen; ra, radius; s, sacral vertebra; sc, scapula; sk, skull; so, supraoccipital; SPDL, spinodiapophyseal lamina; SPOL, spinopostzygapophyseal lamina; SPRL, spinoprezygapophyseal lamina; sr, sacral rib; tc, tibial condyle; ti, tibia; upp, posterior process of unla; tg (V), trigeminal foramen (V); tn (IV), trochlear nerve foramen (IV); un, unla.

### 1.2. Institutional abbreviations

CQ208, HEGT Chongqing Laboratory of Geological Heritage Protection and Research, No. 208 Hydrogeological and Engineering Geological Team, Chongqing Bureau of Geological and Mineral Resource Exploration and Development Chongqing, Chongqing, China; CAGS, Institute of Geology, Chinese Academy of Geological Sciences, Beijing, China; IVPP, Institute of Vertebrate Palaeontology and Palaeoanthropology, Chinese Academy of Sciences, Beijing, China; ZDM, Zigong Dinosaur Museum, Zigong, Sichuan, China.

### 1.3. Method

#### 1.3.1. Terminology

Romanian orientational descriptors (e.g. anterior, posterior) rather than standardized terms (e.g. cranial, caudal) were used. We follow Wilson [40,41] and Wilson *et al*. [42] to employ morphological and orientational descriptors for vertebrae fossae and laminae.

#### 1.3.2. Descriptions and comparisons

All descriptions were made directly from the holotype specimen of *Yuzhoulong qurenensis*. Comparisons with other taxa in this article were made from direct observations of specimens or with published descriptions, illustrations and photographs. For descriptive purposes, the braincase is oriented with the dorsal surface of the occipital condyle horizontally positioned, which is coincident with a horizontal position of the lateral semicircular canal [43]. The exoccipital and opisthotic are fused, and we described them together. The term parabasisphenoid is used to describe the basisphenoid and parasphenoid complex [44]. The scapula and coracoid were described with their long axis orientated horizontally.

#### 1.3.3. Measurements

The length of the deltopectoral crest measures from the distal maximum curvature point of the deltopectoral crest to the proximal-most point.

#### 1.3.4. Phylogenetic analysis

Phylogenetic analyses were carried out in TNT v. 1.5 [45]. Equal weights parsimony (EWP) and extended implied weighting (EIW) analyses are employed in the analyses. A concavity constant (K) of 12 for extended implied weighting was used. The New Technology Search was applied first (xmult = replications 50 hits 10 css rss ratchet 5 fuse 5). Then, the resulting MPTs were used as the starting trees for a Traditional Search using TBR.

## 2. Systematic palaeontology

**Figure 2.**
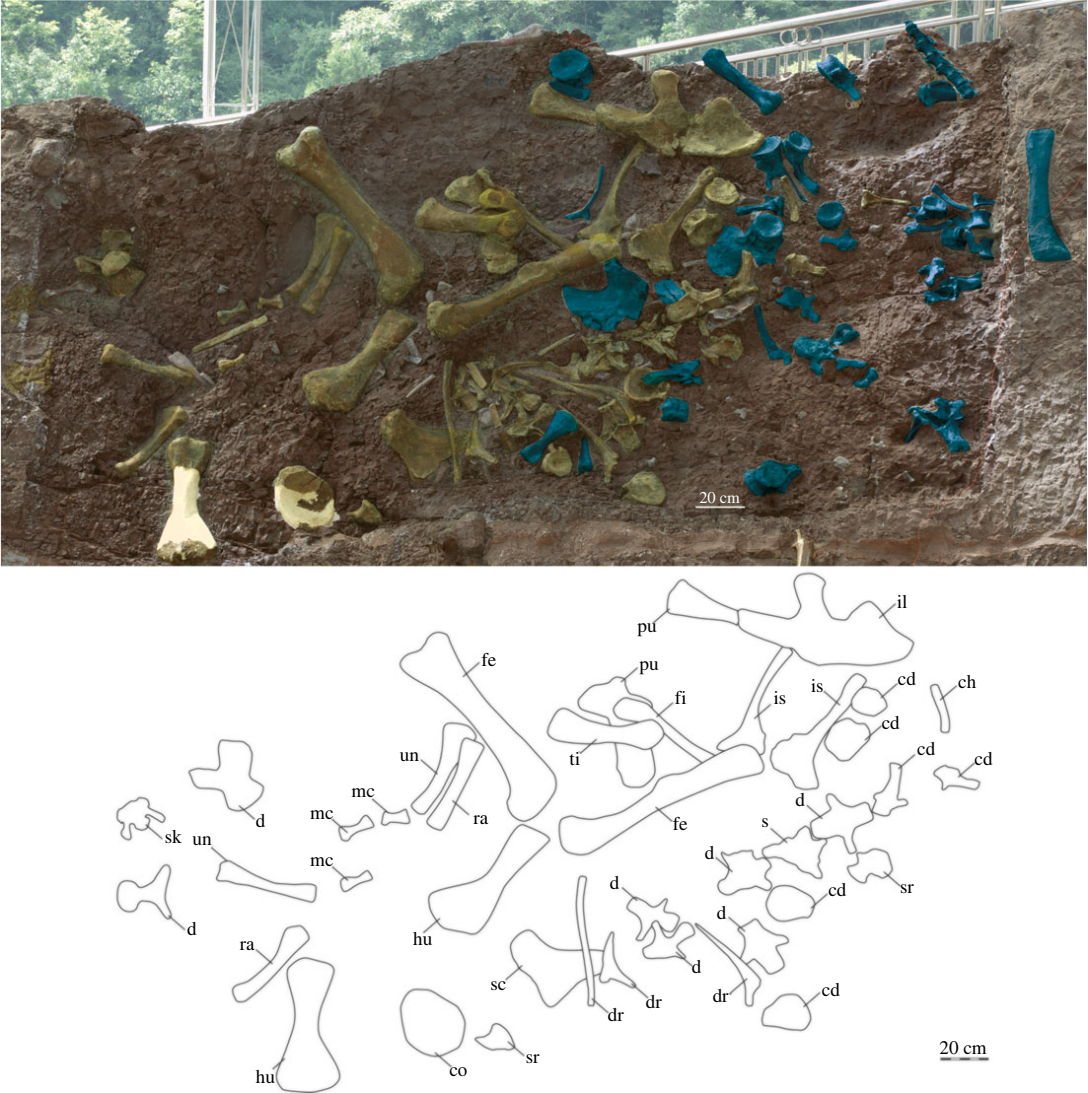
Field sketch of *Yuzhoulong qurenensis*. Materials of *Yuzhoulong* in yellow, another sauropod specimen in blue. cd; caudal vertebra; ch, chevron; co; coracoid; d, dorsal; dr, dorsal rib; fe, femur; fi, fibula; hu, humerus; il, ilium, is, ischium mc, metacarpal; s, sacral vertebra; sc, scapula; sk, skull; sr, sacral rib; ti, tibia; pu, pubis;. ra, radius; un, unla. The scale bar represents 20 cm.

**Figure 3.**
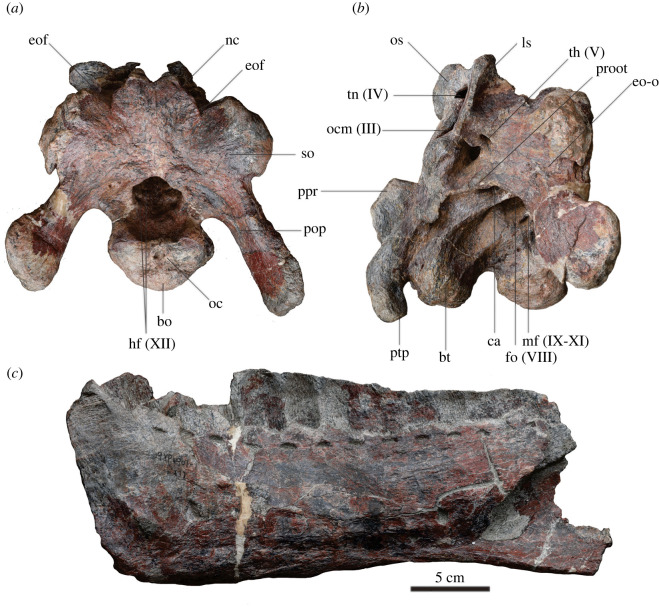
Braincase and dentary of *Yuzhoulong qurenensis*. (*a*), occipital view; (*b*), left lateral view; (*c*), medial view. Abbreviations: bt, basal tuberae; ca, carotid artery; eof, external occipital fenestra for the caudal middle cerebral vein; eo-o, exoccipital-opisthotic complex; fo (VII), fenestra ovalis (VII); hf (XII), hypoglossal foramen (XII); ls, lateraosphenoid; mf (IX-XI), metotic fenestra (IX-XI); nc, nucal crest; ocm (III), oculomotor nerve foramen (III); os, orbitosphenoid; pop, paraoccipital process; ppr, parasphenoid rostrum; proot, prootic; ptp, pterygoid process; so, supraoccipital; tg (V), trigeminal foramen (V); tn (IV), trochlear nerve foramen (IV). The scale bar represents 5 cm.

**Figure 4.**
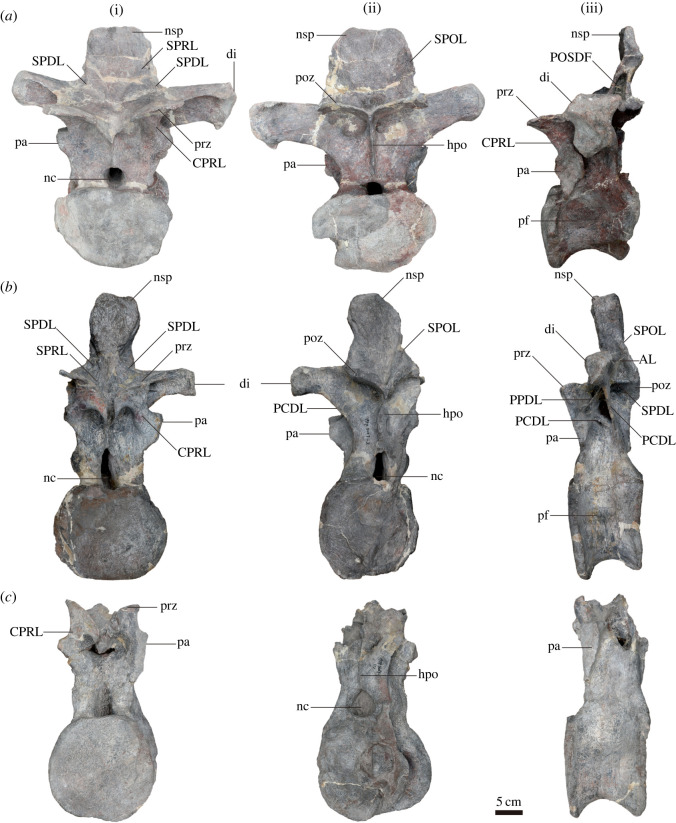
Well-preserved anterior (*a*), middle (*b*) and posterior (*c*) dorsal vertebrae of *Yuzhoulong qurenensis* in anterior (I), posterior (II) and left lateral (III) views. AL, accessory lamina connecting the SPOL and to the diapophysis of middle dorsal vertebrae laterally; CPRL, centroprezygapophyseal lamina; di, diapophysis; hpo, hyposphene-hypantrum system; nsp, neural spine; SPDL, spinodiapophyseal lamina; SPOL, spinopostzygapophyseal lamina; SPRL, spinoprezygapophyseal lamina; pa, parapophysis; PCDL, posterior centrodiapophyseal lamina; pf, lateral pneumatic fossa or foramen; poz, postzygapophysis; POSDF, postzygapophyseal spinodiapophyseal fossa; PPDL, paradiapophyseal lamina; prz, prezygapophysis. The scale bar represents 5 cm.

**Figure 5.**
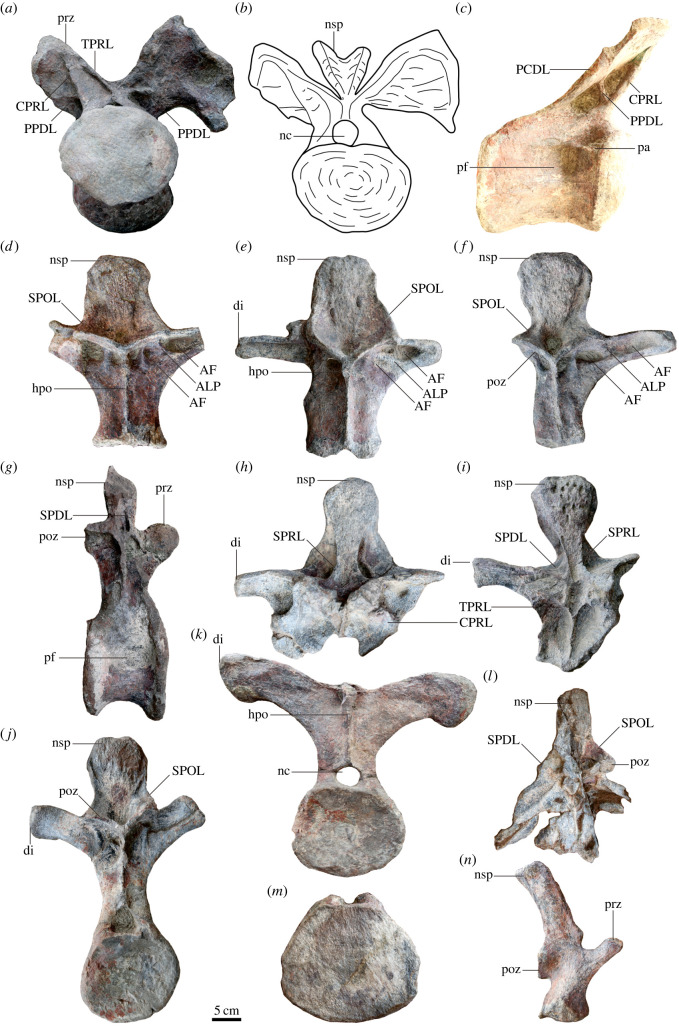
Dorsal (*a*–*k*), sacral (*l*) and caudal (*m*,*n*) vertebrae of *Yuzhoulong qurenensis*. ALP, connects the ACDL to PODL in anterior to middle dorsal neural arches; AS, accessory strut connects to the CPRL and PCDL of anterior dorsal vertebrate; CPRL, centroprezygapophyseal lamina; di, diapophysis; hpo, hyposphene-hypantrum system; nsp, neural spine; SPDL, spinodiapophyseal lamina; SPOL, spinopostzygapophyseal lamina; SPRL, spinoprezygapophyseal lamina; PCDL, posterior centrodiapophyseal lamina; pf, lateral pneumatic fossa or foramen; POSDF, postzygapophyseal spinodiapophyseal fossa; poz, postzygapophysis; pa, parapophysis; PPDL, paradiapophyseal lamina; prz, prezygapophysis. The scale bar represents 5 cm.

**Figure 6.**
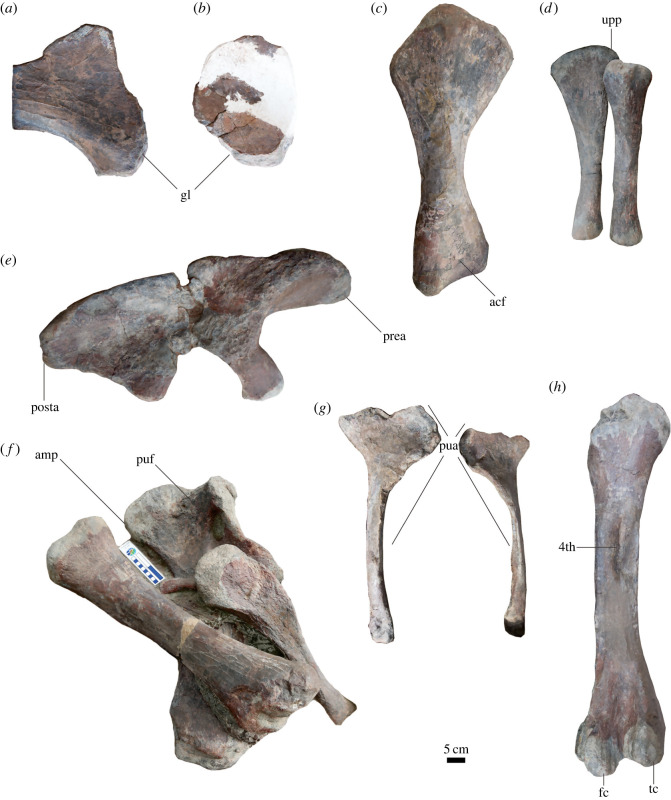
Appendicular elements of *Yuzhoulong qurenensis*. (*a*,*b*), left scapula and coracoid in medial view; (*c*), left humerus in posterior view; (*d*), left unla in posterior view and left radius in anterior view; (*e*), left ilium in medial view; (*f*), right pubis, tibia, and right fibula in anterior view; (*g*), ischia in posterior view; (*h*), left femur in posterior view. 4th, fourth trochanter of femur; acf; anconeal fossa; amp, ambiens process; fc, fibular condyle; gl, glenoid; posta, postacetabular process; prea, postacetabular process; pua, pubic articulation; puf, pubic foramen; tc, tibial condyle; upp, posterior process of unla. The scale bar represents 5 cm.

Dinosauria Owen, 1842
 Saurischia Seeley, 1887
 Sauropodomorpha von Huene, 1932
 Sauropoda Marsh, 1878
 Neosauropoda Bonaparte, 1986
 Macronaria Wilson & Sereno, 1998
 *Yuzhoulong qurenensis* gen. et sp. nov. (figures 2–6)

### 2.1. Holotype

CLGRP V00013. Partly preserved skull, 12 dorsal vertebrae, 1 sacral vertebra, 10 caudal vertebrae, left scapula, coracoid, left and right humerus, ulna and radius, 3 metacarpals, left ilium, left and right pubis, ischia, femur and tibia, left fibula, several dorsal ribs and chevrons (figure 2). This specimen is a sub-mature individual according to most preserved vertebrae are partly preserved.

### 2.2. Etymology

The generic name ‘Yuzhou’ refers to the ancient name of Chongqing in Chinese. ‘Long’ means dragon in Chinese Pinyin. The specific name ‘Quren’ is derived from the name of the ancient Yunyang County.

### 2.3. Diagnosis

A macronarian possessing the following unique combination of character states (autapomorphies are marked by *): Cranial nerve II foramen opens anteriorly and slightly medially divided; anterior, middle to posterior dorsal are opisthocoelous, amphicoelous, respectively; dorsal centra are dorsoventrally compressed; height of neural arches/height of centra is below 1.0 in anterior dorsals, and more than 1.0 in posterior dorsals; two similar sized accessory fossae exist in the posterior surface of dorsal diapophyses of anterior dorsal vertebrae* (figure 5, AF); diapophyses of anterior to middle dorsals are laterally projected, and posterior dorsal diapophyses are dorsolaterally oriented; anterior-most dorsal neural spine is bifurcated, rest of anterior dorsal neural spines are transversely extended with sub-parallel shaped lateral margins, and the distal end of middle to posterior neural spines are prominently transversely extended compare with the bottoms; the distal surfaces of anterior dorsal neural spines are flat, and that of middle to posterior dorsal neural spines are convex; an accessory lamina (AL) connecting the spinopostzygapophyseal lamina (SPOL) and to the diapophysis of the middle dorsal vertebrae laterally (figure 4*b*, AL) anterior caudal centra are amphicoelous and dorsoventrally compressed; proximodistal length of humeral shaft/femoral shaft is less than 0.6; preacetabular process of ilium is prominent dorsolaterally twisted, making the process nearly perpendicular to the iliac blade* (figure 6*e*).

### 2.4. Locality and horizon

The materials were excavated in Laojun Village, Pu'an Town, Yunyang Country, Chongqing Municipality, Southwest China (figure 1). Sauropod remains were found in purplish-red silty mudstones located in the middle portion of the Lower Shaximiao Formation. The age of the Lower Shaximiao Formation was inferred as Bathonian to Callovian age traditionally (e.g. [46,47]). Although a series of detrital zircon U-Pb geochronology for this formation in the Sichuan Basin was carried on, the accurate age for this formation is still controversial (e.g. [15–18]). Recently, a new zircon U-Pb age from Yunyang geochronology yielded a maximum depositional age of 166.0 ± 1.5 Ma (late Middle Jurassic) below the bone-bearing horizon of the Lower Shaximiao Formation [19]. In general, we suggest the bone-bearing horizon may belong to the Bathonian.

## 3. Description and comparison

### 3.1. Skull and mandible

Several bones of the braincase and the right dentary are preserved (figure 3). Stapes are missing in the braincase and occiput region, as in most of the sauropod taxa [44]. The supraoccipital is a massive single bone that forms the posterior roof of the endocranial cavity. The well-marked notch, vertically situated at the middle portion of the dorsal surface, articulates with the parietal, which gives support to the ventral process of the parietal. The external occipital fenestra for the posterior middle cerebral vein [48] is present in the deepest part of the notch, which opens internally into the brain cavity. The supraoccipital is highly fused with the prootic that forms the ventral support for the parietal, laterodorsally. It differs from that in *Europasaurus* with a narrow but well-developed nuchal crest [44].

The exoccipital and opisthotic are fused in *Yuzhoulong qurenensis*. This condition also is observed in *Europasaurus* [44]. No prominent trace above the metotic fissure marks the line of union of these two bones, similar to that in *Europasaurus* [49]. It differs from that of *Rapetosaurus* with a slight trace [49]. The suture of the exoccipital–opisthotic complex with the underlying basioccipital is invisible in occipital and lateral views. The paroccipital process (figure 3, pop) is ventrolaterally directed, at an angle that is higher than in *Camarasaurus* [50]. The hypoglossal nerve shares two foramina at the basal portion of the exoccipital–opisthotic complex that connects to the basipterygoid. This condition is similar to many basal sauropods [51], whereas it differs from that in derived sauropod taxa such as *Camarasaurus* [50].

The prootic is generally fused with the adjoining bones on its posterior, ventral and dorsal sides. The anterior end articulates with the laterosphenoid, the ventral portion contacts the parabasisphenoid and the posterior portion articulates with exoccipital–opisthotic complex and supraoccipital. The basioccipital, situated at the base of the foramen magnum, has a minor contribution to it. Most of the dorsal surface of the basioccipital articulates the exoccipital. A narrow sulcus could be observed in the conjunction between the basioccipital and exoccipital, a product of the extensive lateromedial ventral expansion of the exoccipital, as occurred in some camarasauromorphs such as *Camarasaurus* and *Giraffatitan* [50,52]. In the occipital view, the occipital is crescent-shaped, and the width is about 1.41 the height. This ratio is generally similar to that in *Limaysaurus tessonei* (MUCPv 205: 1.37) and *Europasaurus* (1.5), but greater than that of most neosauropods [53]. A deep fossa exists on the ventral surface between the basal tubera and the basipterygoid fossa. This condition is widespread in macronarians that are more derived than *Camarasaurus* [54]. The width of the basal tubera is about 1.2 the width of the occipital condyle, generally similar to *Limaysaurus tessonei* (MUCPv 205: 1.37), *Diplodocus longus* (USNM 2673, 1.26), and *Camarasaurus lentus* (CM 11338, 1.32) [53].

In ventral view, the parabasisphenoid forms a deep medial fossa that contacts the basioccipital. The basipterygoid processes are formed by the parabasisphenoid ventrally with a deep anteroposteriorly extended fossa, and the two processes generally form a 45° angle, similar to that in the basal tubera. The basipterygoid processes are mediolaterally compressed, about perpendicular to the horizontal plane when the braincase is positioned in its presumed neutral position horizontally. The distal end of the process is slightly deformed during the preservation which makes it posteriorly projected in lateral view. Cranial nerve II foramen opens anteriorly, located at the centre of the orbitosphenoid. This foramen is slightly medially divided, as occurred in *Suuwassea* and *Europasaurus* [44,55,56]. Note that cranial nerve II foramen is not medially divided but instead forms a single anterior foramen, as described for *Shunosaurus* [57], and *Mamenchisaurus* [58], but the division is much-limited compared with other later diverged sauropods such as *Amargasaurus*, *Camarasaurus* and *Giraffatitan* [50,52,59].

The preserved right dentary is incomplete with the posterior-most portion missing, but all alveoli are completely preserved. As in other sauropod taxa, the articulated dentaries share a U-shaped morphology in dorsal or ventral view. According to the preserved portion, the angular articulation of the dentary situated at the posterior end of the dentary with a broad channel-like concave surface extends posterodorsally from around a fourth of the dentary up to the dorsal surface laterally. A well-developed, V-shaped Meckelian canal originates posteriorly from the dorsal and ventral rami and shallowly anteriorly extended up to the level of about the eighth alveolus. This fossa enhances the strength to support the prearticular and the splenial, similar to other sauropods. The preserved dentary has 13 alveoli, but the teeth are not preserved. This condition is similar to that of some derived camarasauromorphs such as *Camarasaurus* (12 or 13), and *Euhelopus* (13) [50,60,61]. It differs from some non-neosauropod taxa of the Lower Shaximiao Formation (e.g. *Shunosaurus lii* (14 or 15)).

### 3.2. Dorsal vertebrae

Twelve dorsal vertebrae are isolated and preserved in the quarry (figures 4 and 5, table 1 for measurements). Most of the dorsal vertebrae are partly preserved with most parts still preserved in the location. Only a few views could be observed from most of them. D1 is partly extracted with the posterior portion still buried in the field. D2, D7, and D10 are completely excavated, but only D2 is well preserved. The D10 was excessively repaired, making the internal pneumatic cavity observable in the anterior and lateral views. D3, D4, and D7 are partly preserved with neural arches and neural spines explored. D7 is partly observed with the right view and the right diapophysis is missing. D11 is generally complete with the posterior surface visible. Only neural arch and centrum are preserved with posterior surface observable. The exact number of dorsal vertebrae of *Yuzhoulong qurenensis* is unknown. According to the preserved feature and number of dorsal vertebrae, the actual number of dorsal vertebrae is more than 12. This condition may be similar to that in some macronarians (e.g. *Euhelopus zdanskyi*) with 13 dorsal vertebrae [60]. Rather than fully describing the anatomical features of vertebrae, we described the preserved dorsal series in one part and documented changes along the vertebral sequence. Dorsal vertebrae (D) 1 and 3 are presumed as the anterior dorsal vertebrae according to the parapophysis is still in contact with the centrum; the preserved centra are opisthocoelous (referred [54,62]) and the postzygapophyses are generally horizontally extended. D4 to D8 are defined as the middle dorsal vertebrae considering the zygapophyses are about 45° to horizontal; the diapophysis processes are laterally projected. The rest of the preserved dorsal vertebrae (D9 to D12) are numbered as the posterior dorsal vertebrae according to the diapophyses processes are dorsolaterally extended; the parapophyses are located near the same level as the prezygapophyses. According to the preserved dorsal vertebrae, the centra of the dorsal vertebrae are solid.

**Table 1.** Measurements of vertebrae of *Yuzhoulong qurenensis*. ACH, anterior centrum dorsoventral height; ACW, anterior centrum transverse width; CD, caudal vertebrae; CLB, centrum length (including ball); D, dorsal vertebrae; DFA, distance from anterior end of centrum to anterior margin of neural arch; DFP, distance from posterior end of centrum to posterior margin of neural arch; NAH, neural arch dorsoventral height (measured from dorsal margin of centrum up to the base of the postzygapophyses); NSH, neural spine dorsoventral height (measured from base of postzygapophyses up to neural spine summit); NSL, neural spine maximum anteroposterior length (measured above SPOLs); NSW, neural spine maximum transverse width; PCH, posterior centrum dorsoventral height; PCW, posterior centrum transverse width; Vn, vertebrae number (the number of dorsal and caudal vertebrae only presents the sequence of vertebrae).

Vn	CLB	ACH	ACW	PCH	PCW	DFA	DFP	NAH	NSH	NSL	NSW
D1	204	125	179		201	50		94^a^			
D2	168	141	199	139	204	53	32	126	146	2	122
D3								126^a^	157		92
D4								143^a^	185		92
D5								145^a^	150		104
D6	150	185		166							
D7	153	194	215	192^a^	220^a^	19	28	151	214	67	104
D8									196	49	90
D9									179		98
D10	150	180	185	176	174^a^	33	16	160^a^			
D11				172	182			186	172		
D12				170	195			170			
CD1				180	216						
CD2				205	222	13					
CD3				143	174						
CD4	96			210	225						
CD5	92			132^a^	223						
CD6	68^a^			165	189^a^						
CD7	113			162	180						
CD8		135	147								
CD9	80			170	175						
CD10	88			138	140						

^a^denotes a measurement based on an incomplete element.

The centrum of anterior dorsal vertebrae is opisthocoelous with a prominently extended anterior condyle (figures 4*a* and 5*a–c*). By contrast, the middle to posterior dorsal centra are amphiplatyan and amphicoelous (figures 4*b*,*c* and 5*k*,*j*). This condition is similar to some early diverging eusauropods (e.g. *Shunosaurus* and *Patagosaurus*) and some diplodocids such as *Lingwulong* [61,63,64]. Both anterior and posterior articular surfaces are dorsoventrally compressed, as occurred in most other macronarians [65]. The ventral surfaces of the centra are transversely convex and concave anteroposteriorly. The midline keel only exists in D1, and two shallow concavities are situated on both sides of this middle convexity. By contrast, the midline keels are present till D4 in *Dashanpusaurus dongi* [34]. The ventrolateral ridge is absent on the ventral surfaces of the centra. The lateral pneumatic fossa is elliptical-shaped in outline with no septum divided, similar to that in many early diverging macronarians [66,67]. The parapophyses are located at the middle portion dorsoventrally near the anterior articular condyle in D1, then ascent in subsequent dorsal vertebrae with the bottom portion of D2 extended from the centrum and located between centrum and prezygapophyses in the middle dorsal vertebrae, finally nearly at the same level with the articular surface of prezygapophysis of posterior dorsal vertebrae.

According to the preserved dorsal elements, the neural arch is shorter than the centrum in the anterior dorsal neural arches, whereas the height of the neural arch/height of the centrum is larger than 1.0 in posterior dorsal vertebrae. This condition differs from that in *Dashanpusaurus dongi* with a ratio of approximately 1.1 to 1.2 in the whole dorsal vertebrate series [34]. In anterior dorsal vertebrae, the prezygapophyses are dorsally oriented with the articular surface generally parallel to the horizontal. By contrast, prezygapophyses are approximately 45° to horizontal in the middle and posterior dorsal vertebrate series. The prezygapophyses extend beyond the centra and are supported by the transversely thin undivided CPRL. The diapophyses are robust and situated on the dorsal margin of the neural arches. The articular surfaces of the diapophyses are sub-triangular shaped in outline, and slightly concave. Diapophyses of anterior to middle dorsal vertebrae are laterally extended, and these processes of posterior dorsal vertebrae are dorsolaterally projected. A dorsolaterally oriented middle to posterior diapophysis is similar to that in many early diverging sauropods such as *Shunosaurus lii*, and *Cetiosaurus oxoniensis* [61,68], but these dorsolaterally projected diapophyses are present in middle to posterior dorsal vertebrae, rather than limited in posterior dorsal vertebrate series. Accessory lamina (figure 5*d–f*, ALP), dorsomedially extended, connects the PCDL to PODL in anterior to middle dorsal neural arches. The POCDF of anterior to middle dorsal vertebrae are divided by this lamina into two shallow fossae. PPDL of D1 is a thin lamina, extended from the base of CPRL, that connects to the CPRL and PCDL. The hyposphene–hypantrum system is prominent. It is rather a robust convexity that is situated on the dorsal margin of the neural canal. In preserved dorsal vertebrae (D2, D7 and D10), the PRCDF and CDF are many deep concavities situated on dorsal neural arches. These are divided by the PPDL. According to D10 (figure 4*c*), the internal pneumatic cavity exists in the posterior dorsal vertebrae but lacks the external opening on the neural arch. It differs from that in some early diverging non-neosauropod eusauropods (e.g. *Patagosaurus*) and *Dashanpusaurus* with internal cavities and lateral openings on the middle/posterior dorsal neural arch [34,63].

The neural spine of D1 is bifurcated, whereas the rest of the preserved dorsal neural spines are non-bifurcated. The bifurcated condition is similar to some macronarians (e.g. *Camarasaurus*, *Bellusaurus*, *Lourinhasaurus* and *Opisthocoelicaudia*) [69–71], and some non-neosauropod eusauropod taxa such as *Mamenchisaurus* [57]. The neural spine of D1 is shallowly bifurcated, as occurred in *Dashanpusaurus*, *Bashunosaurus* and *Bellusaurus* [30,34,67], whereas it differs from the more strongly bifurcated taxa with the bifurcation beginning from the basal portion of the neural spine of the anterior dorsal vertebrae (e.g. *Camarasaurus*) and usually with the medial process occupied between the two metapophyses (e.g. *Apatosaurus*) [69,72]. A strongly limited number of bifurcated dorsal vertebrae (only anterior-most dorsal vertebra share a bifurcated neural spine) of *Yuzhoulong qurenensis* is different from other bifurcated taxa that share with more than one bifurcated dorsal neural spine. The neural spines are anteroposteriorly compressed throughout the rest of the non-bifurcated anterior dorsal vertebrae series and have a thick, plate-like appearance in outline. By contrast, the middle dorsal neural spine is transversely extended distally compared with the bottom portion. This condition differs from that in *Bashunosaurus* with a prominently convex distal end in the middle dorsal neural spine [30]. SPRL and SPDL are extended to the middle portion of neural spines. By contrast, most of the SPOLs are approximately extended to the summit of neural spines. Moreover, an accessory lamina (AL) connects the SPOL and to the diapophysis of the middle dorsal vertebrae in lateral view (figure 4*b*III, AL). This thin lamina dorsoposteriorly extends from the dorsoposterior margin of diapophysis to the middle portion of SPOL.

### 3.3. Dorsal ribs

At least two dorsal ribs are partly preserved. Two proximal portions and one distal portion are visible (figure 2). The proximal part of the rib shaft is directed ventrally and slightly laterally relative to the vertically oriented tuberculum. According to the preserved portion, the cross-section of the ribs is elliptical-shaped. No coel exists on the preserved proximal rib head. The internal structure of the rib is not known.

### 3.4. Sacral vertebrae

One sacral vertebra has been partly preserved with the bottom of the neural arch and the centrum missing (figure 5*l*). We suggest this vertebra from the sacral vertebrate series for the following reason: the diapophysis of this vertebra is dorsoventrally projected, and this condition is much different from that of dorsal vertebrate series with laterally or dorsolaterally oriented diapophyses. The diapophysis of this vertebra is more robust than any other diapophyses of dorsal vertebrae with SPDL prominently laterally extended dorsally to enhance the mechanical strength of diapophyses. It may be the caudosacral vertebra of sacral series. The preserved height of the neural arch is about equal to the height of the neural spine. As mentioned above, the diapophyses are robust with the basal portion distinctly dorsoventrally extended. The postzygapophyses are generally ventrally oriented with the articular surfaces approximately parallel to the horizontal.

### 3.5. Caudal vertebrae

Ten caudal vertebrae (Cd) are incomplete with the centrum preserved and most of the portions still buried in the quarry (figure 5*m*, table 1 for measurements). Additionally, the other four vertebrae are partly preserved with centra missing (figure 5*n*). According to the morphology (e.g. the size of these centra), these caudal can be identified as anterior caudal vertebrae. The centra of all preserved caudal vertebrae are amphicoelous. This condition resembles *Camarasaurus lewisi* and most of the early diverging sauropod taxa such as *Shunosaurus lii* and *Omeisaurus tianfuensis* [61,66]. By contrast to the two articular surfaces of the caudal centra, the anterior articular surfaces are much more concave than the posterior ones. All the preserved caudal vertebrae are dorsoventrally compressed, similar to most macronarians (e.g. *Bellusaurus*) and many non-neosauropod eusauropods such as *Cetiosaurus oxoniensis* [65]. This condition differs from that in *Dashanpusaurus* with most of the anterior centra being transversely compressed. The ventral surface of the caudal centra is transversely convex and anteroposteriorly concave with no excavations or ridges. The lateral pneumatic fossa is absent in the preserved caudal series.

In lateral view, the prezygapophyses are steeply inclined anterodorsally on the anterior neural arches of preserved caudal vertebrae. Lamination of the caudal vertebra is poorly developed, similar to that in many macronarians. PRDL, SPRL and SPOL exist in anterior caudal vertebrae. The neural spines are vertically and slightly posteriorly projected, with an anteroposterior length larger than the transverse width. The distal ends of neural spines are transversely convex and flat anteroposteriorly with an irregularly flat central portion in the anterior view.

### 3.6. Chevrons

At least two anterior chevrons are preserved (figure 2). The expanded proximal ends of the haemal arches form a continuous bridge of bone over the haemal canal. This condition is similar to many early diverging sauropods (e.g. *Omeisaurus tianfuensis*). Both left and right articular surfaces are mildly concave transversely and convex anteroposteriorly, with the long-axis of the haemal arch held vertically. In dorsal view, the anterior parts of the articular surfaces are smaller than the posterior parts in the anterior ones. The haemal canal of anterior chevrons is a dorsoventally elongated ellipse in anterior or posterior view. The left and right rami are transversely compressed with slightly convex medial surface and more strongly convex lateral surfaces. Laterally, the distal blade of each anterior chevron is broad and rounded. It narrows transversely with a slightly posteriorly curved distal tip.

### 3.7. Scapula and coracoid

Only the anterior portion of the left scapular is partly preserved, with the medial surface of the scapular acromion and the anterior portion of the scapular blade visible (figure 6, table 2 for measurements). According to the observable portion, the scapular blade is ‘D’-shaped, similar to many sauropod taxa such as *Camarasaurus* [66]. The acromion process is a slightly dorsally expanded plate, with acute dorsal expansion. In lateral view, the coracoid articulation site is about 70° to the long axis of the scapular blade, whereas that in *Dashanpusaurus* is generally perpendicular to the long axis. The scapular glenoid surface is slightly excavated and anterolaterally oriented. The medial surface of the expanded proximal portion of the scapular blade is flat, and increasingly dorsoventrally expanded near the scapular acromion naturally.

**Table 2.** Measurements of pectoral girdle and forelimb of *Yuzhoulong qurenensis*. HRI: the average of the greatest widths of the proximal end, mid-shaft and distal end of humerus/length of humerus.

element	dimension	measurement
scapular (left)	length of glenoid surface along its long-axis	241
	transverse width of the glenoid (measured at the junction with the coracoid)	158
coracoid (left)	anteroposterior length on lateral surface	299
humerus (left)	length	734
	proximal end maximum mediolateral width	323
	proximal end maximum anteroposterior width	111
	mediolateral width at midshaft	120
	anteroposterior width at midshaft	58^a^
	distal end maximum mediolateral width	217
	hri	0.3
humerus (right)	length	727
	proximal end maximum mediolateral width	237^a^
	mediolateral width at midshaft	122
	distal end maximum mediolateral width	0.27
ulna (left)	length	508
ulna (right)	length	510
radius (left)	length	505
	proximal end maximum mediolateral width	126
	proximal end maximum anteroposterior width	75
radius (right)	length	500
	proximal end maximum mediolateral width	124
	proximal end maximum anteroposterior width	85
	anteroposterior width at midshaft	47^a^
metacarpal i (left)	length	135
	maximum width of proximal end	65^a^
	maximum width of disatal end	66
metacarpal ii (left)	length	170
	maximum width of proximal end	82
	maximum width of mid-shaft	40
	maximum width of disatal end	70
metacarpal v (left)	length	150
	maximum width of proximal end	70
	maximum width of mid-shaft	35
	maximum width of distal end	53

^a^denotes a measurement based on an incomplete element. All measurements are in millimetres.

The left coracoid is partly preserved with the middle portion restored by plaster and only the medial surface is observable (figure 6). The most robust portion of the coracoid is situated at the glenoid. It is transversely narrow from the bottom to the dorsal distal. The medial surface of the coracoid is concave, and the deepest portion is located at the centre. The articular surface of the glenoid is the sub-triangular-shaped outline. The anterodorsal margin of the coracoid is sub-rounded, as occurred in many sauropod taxa (e.g. *Camarasaurus*) [66]. The coracoid foramen is not preserved. In the lateral view, the dorsal and anterior margins of the coracoid merge smoothly. The infraglenoid lip of the coracoid is absent, resembling that in most macronarians such as *Camarasaurus* [66].

### 3.8. Humerus

The left and right humeri are well-preserved and the middle portion of the right humerus is restored, only posterior surfaces could be observed with most other portions buried (figure 6, table 2 for measurements). The HRI (the average of the greatest widths of the proximal end, mid-shaft and the distal end of the humerus/proximodistal length of the humerus) values are 0.30 (left) and 0.27 (right), respectively. These ratios are similar to most other eusauropods [65]. In posterior view, both proximal and distal ends are transversely extended compared with the middle portion. The proximal width is 0.44 (left) the total length of the humeral shaft, greater than that in *Camarasaurus lewisi* (36%) and *Lourinhasaurus alenquerensis* (39%) [66,70]. According to the preserved portion, the cross-section of the mid-shaft is elliptical, similar to most other macronarians (e.g. *Bellusaurus*) [67]. The shape of the humeral distal end is quadrilateral. The two condyles are not divided with slightly convex coarse surfaces. The distal width is 0.30 (left) and 0.32 (right) proximodistal length of the humerus, respectively.

### 3.9. Ulna and radius

The two ulnas are well preserved, but only posterior surfaces are observable (figure 6, table 2 for measurements). The ulna is slightly longer than the radius, and the ulnar length is about 1.01 (left) the total length of each radius. The total proximodistal length of the ulna is about 0.70 the proximodistal length of each humerus. The posterior process and the olecranon process are weakly developed. The expanded proximal end is transformed into a sub-circular cross-section at mid-shaft, as occurred in most other sauropods such as *Camarasaurus lewisi* [66]. The distal surface of the ulna is an elliptical-shaped outline.

Two radii are well preserved. Only anterior surfaces are visible (figure 6). The length of each radius is 0.69 the length of each side of the humerus. This ratio is generally similar to some macronarians such as *Camarasaurus lewisi* (0.71) and *Lourinhasaurus alenquerensis* (0.73), but bigger than that in *Bellusaurus sui* (0.60) [66,67,70]. The maximum length of the long-axis in the proximal end is 0.25 of the total radial length, as in *Lourinhasaurus alenquerensis* (0.25) and *Camarasaurus lewisi* (0.24) [66,70]. The proximal end is an elliptical shaped outline with the anterior margin distinctly extended. The articular surface of the proximal end is nearly perpendicular to the long axis of the shaft, similar to that in *Camarasaurus lewisi* and *Bellusaurus sui* [66,67]. The distal articular surface of the radius is an irregularly comma-shaped outline. Similar to the proximal end, the distal end is nearly perpendicular to the long axis of the shaft with a rugose articular surface.

### 3.10. Metacarpals

Three left metacarpals are well preserved with most portions still buried in the quarry (figure 2). These three elements are defined as the Mc. I, Mc. II and Mc. V according to the proximal and distal articular facts. Mc. I is a robust and rod-like element with expanded proximal and distal ends. In ventral view, the proximal end is prominently transversely extended. The distal condyle of Mc. I is divided. The lateral portion of the distal end is distinctly laterally extended, which makes the transverse axis of the distal condyle of Mc. I bevelled about 20° with respect to the axis of the shaft. This condition is similar to many other eusauropod taxa (e.g. *Omeisaurus tianfuensis*). The length of Mc. II is larger than the other two metacarpals. Both proximal and distal ends are prominently expanded. The shaft is slightly twisted which means the long-axis of proximal and distal ends are generally not on the same plane. The Mc. V is a slender element with prominently laterally extended proximal end. The outline of the proximal surface is sub-triangular.

### 3.11. Ilium

The left ilium is completely preserved (figure 6, table 3 for measurements). Laterally, the dorsal margin of the ilium is semicircular shaped, as occurred in almost all eusauropods such as *Camarasaurus lewisi* and *Bellusaurus sui* [66,67]. The preacetabular process projects anterolaterally, beyond the anterior end of the pubic peduncle in lateral view, resembling most eusauropods such as macronarians (e.g. *Camarasaurus lewisi*) [66]. By contrast to that in *Dashanpusaurus*, the preacetabular process projects much more laterally in *Yuzhoulong qurenensis*, and the transverse width of the dorsal portion of preacetabular in *Yuzhoulong qurenensis* is much thicker than that in *Dashanpusaurus*. It indicates that *Yuzhoulong qurenensis* shares a more robust and laterally projected preacetabular process. The preacetabular process of the ilium is prominent dorsolaterally twisted, making the process nearly perpendicular to the iliac blade (figure 6*e*). This condition differs from other eusauropod with the preacetabular process of the ilium laterally projected, rather than prominently dorsolaterally twisted (e.g. [73]). The highest point of the iliac dorsal margin is situated anterior to the base of the pubic peduncle, similar to many other neosauropods such as *Euhelopus zdanskyi* [60]. The angle between the ventral surface of the preacetabular process and the anterior face of the pubic peduncle is about 90°, as occurred in *Dashanpusaurus dongi*, *Bellusaurus sui*, and many other eusauropods (e.g. *Cetiosaurus oxoniensis*) [34,67,68]. The distal end of the postacetabular process is rounded laterally. The pubic peduncle curves slightly anteroventrally with a mildly convex anterior surface. By contrast, the ischial peduncle is prominently reduced, as in *Camarasaurus lewisi*, *Bellusaurus sui*, and most gravisaurians [64,66,67].

**Table 3.** Measurements of pelvic girdle and hindlimb of *Yuzhoulong qurenensis*.

element	dimension	measurement
ilium (left)	length (between the tips of the anterior and posterior lobes)	960
	length of anterior lobe (from the tip of this lobe to the base of the pubic peduncle)	320
	length of posterior lobe (from the tip of this lobe to the base of the ischiac peduncle)	201
	length of pubic peduncle	220
	width of preacetabular	73
	width of mid-portion of iliac blade	30
	width of postacetabular	48^a^
	length of ischiac peduncle	110
	height of the iliac blade above the pubic peduncle	274
	diameter of acetabulum between the pubic and ischiac peduncles	248
pubis (left)	length (from distal end to the point where the iliac articulation meets the acetabular margin)	710
	length of iliac articulation along its lateral edge	165
	maximum length of the acetabular surface	135
	maximum transverse width of the acetabular surface	82
	maximum transverse width of the ischiac articulation	80
	maximum diameter of obturator foramen	65
	anteroposterior diameter of distal end	215^a^
	maximum transverse width of distal end	90^a^
ischium (left)	dimension preserved length	640^a^
	maximum mediolateral width of iliac peduncle	60^a^
	dorsoventral height of pubic articulation	368^a^
	anteroposterior length of proximal plate	280
	minimum dorsoventral height of ischial blade	71
	maximum dorsoventral height of ischial blade (at distal end)	90^a^
ischium (right)	dimension preserved length	730
	anteroposterior length of iliac peduncle	105
	maximum mediolateral width of iliac peduncle	50^a^
	length of iliac articulation along its lateral edge	140
	dorsoventral height of pubic articulation	434
	anteroposterior length of proximal plate	298
	maximum dorsoventral height of ischial blade (at distal end)	110^a^
	maximum mediolateral width of ischial blade (at distal end)	65
femur (left)	length	1080
	maximum anteroposterior width of the proximal head	100^a^
	transverse width of proximal end	240^a^
	distance from the proximal end of the femur to the top of the 4th trochanter	570
	anteroposterior length of mid-shaft	52^a^
	transverse width of mid-shaft	125
	anteroposterior length of distal end	72
	transverse width of distal end	268
	anteroposterior width of the tibial condyle	165
	anteroposterior width of fibular condyle	170
femur (right)	length	1080
	transverse width of proximal end	288
	transverse width of mid-shaft	131
	anteroposterior length of distal end	80
	transverse width of distal end	294
	anteroposterior width of fibular condyle	140^a^
tibia (left)	length	660
	transverse width of mid-shaft	91
	transverse width of distal end	155
	anteroposterior width of distal end	135
tibia (right)	maximum transverse diameter of the proximal end	234
	transverse width of mid-shaft	93
fibula (left)	length	620^a^
	maximum anteroposterior diameter of the proximal end	140
	maximum transverse diameter of the proximal end (including cnemial crest)	65
	anteroposterior length of mid-shaft	53

^a^denotes a measurement based on an incomplete element. All measurements are in millimetres.

### 3.12. Pubis and ischium

The left and right pubes are well preserved with the lateral surface of the left pubis and the lateral surface of the right pubic distal portion being observable. The remaining portions of each pubis are still buried in the quarry (figure 6, table 3 for measurements). The ischial articulation is medially extended with an ‘S’-shaped outline. The pubic foramen is situated on the upper portion of the shaft, located below the acetabular articular surface, and between the ischial and iliac articulations. It is elliptically shaped with the long axis dorsomedially extended. The distal end is anteroposteriorly extended. The distal end is elliptical-shaped in outline with an irregular concavity on the distal surface.

Left and right ischia are almost completely preserved and not fused with the anterior surface visible (figure 6*g*). The proximal end is expanded anteroposteriorly. Both iliac and pubic articular surfaces are generally elliptical shaped outlines. There is no tuberosity at the medial surface of the iliac articular process. The length of the pubic articular surface is more than 0.5 the total proximodistal length of the ischial shaft (figure 6, pua). This condition is similar to other neosauropod taxa such as *Camarasaurus lewisi* [65,66]. The distal end of the ischial blade is a bladelike-shape outline, without prominent anteroposterior extension.

### 3.13. Femur

The left and right femurs are well-preserved with the posterior surface of the left one and the anterior surface of the right one visible (figure 6, table 3 for measurements). The femoral head projects medially, similar to many sauropods such as *Camarasaurus lewisi*, and *Bellusaurus sui* [66,67]. A lateral bulge (defined as the lateral expansion and a dorsomedial orientation of the dorsolateral margin of the femur (refer to [74])) is absent, similar to that in *Camarasaurus lewisi*, *Bellusaurus sui*, and many other neosauropod taxa (e.g. *Apatosaurus louisae*), whereas that in *Dashanpusaurus* is present [34,66,67,75]. The anterior surface is smooth without ridge, whereas two ridges exist in that of *Dashanpusaurus*. The shaft is a transversely expanded ellipse in cross-section throughout most of its length except the proximal and distal ends. The fourth trochanter is situated on the posteromedial margin of the shaft. This condition is similar to *Camarasaurus lewisi* and many other sauropod taxa [66]. The fourth trochanter is invisible in the anterior view. The tibial condyle is anteroposteriorly larger than the fibular condyle in distal view. The articular surfaces of the two condyles are mildly rough with irregular concavities. The distal articular surface is generally perpendicular to the femoral shaft and resembles *Camarasaurus lewisi* [66].

### 3.14. Tibia and fibula

The left and right tibiae are well preserved with the posterior surface of the proximal portion of the left tibia and the anterior surface of the right tibia observable (figure 6, table 3 for measurements). The total length of the tibia is about 0.61 the total length of the femur. This ratio is similar to that in *Dashanpusaurus* and within the typical range for sauropods [34,66,76]. The shaft of the tibia is transversely wider than anteroposterior length, as occurred in *Camarasaurus lewisi*, *Bellusaurus sui*, and many other eusauropods [66,67]. According to the observed proximal portion, the cnemial crest is robust. It projects laterally and slightly posteriorly to articulate with the anteromedial surface of the fibula. The cross-section of the mid-shaft is an elliptical-shaped outline with a transverse width of 0.14 the total length of the shaft and 0.59 of the transverse width of the distal end. These ratios are generally near to these of *Dashanpusaurus dongi* (0.28 and 0.65) and *Bellusaurus sui* (0.28 and 0.72). The anteroposterior length of the distal end is greater than the transverse length with a ‘comma’-shaped outline, which resembles *Camarasaurus lewisi*, and *Bellusaurus sui* [66,67].

Only the medial surface of the anterior portion of the left fibula is visible (figure 6). In medial view, the fibula is a rod-like shaped slender shaft with a slightly sigmoid outline with the proximal end mildly posteriorly projected. The tibial scar is prominent on the medial face of the proximal portion of the fibula shaft. The articular surface of the tibial scar is slightly concave in medial view.

## 4. Phylogenetic analysis

**Figure 7.**
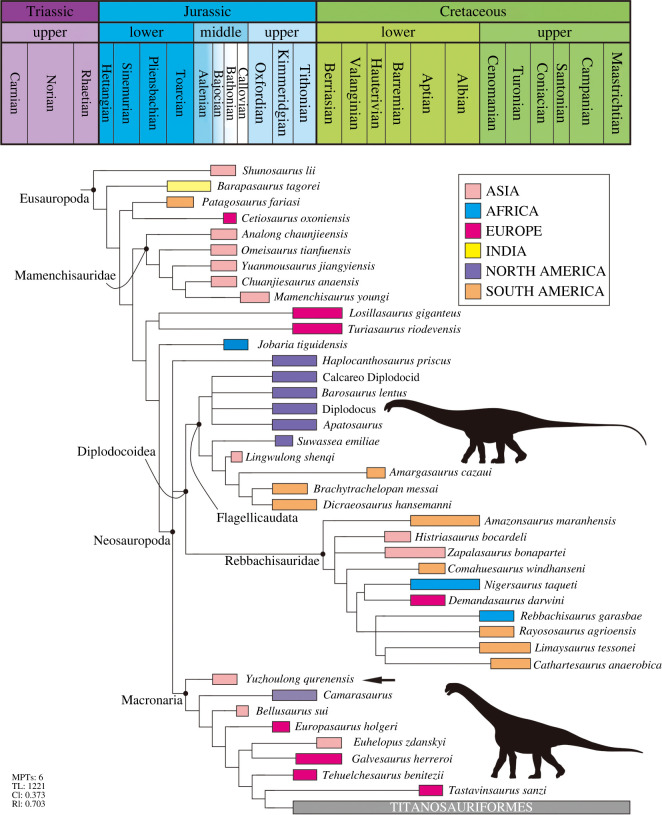
Strict consensus tree of EWP analysis from the main data matrix.

Phylogenetic analyses were conducted to assess the affinities of *Yuzhoulong qurenensis* within Macronaria (figure 7). To test the hypothesis that *Yuzhoulong qurenensis* represent an early diverging macronarian, we have scored the specimen for the data matrix of Ren *et al.* [65] (the main matrix, electronic supplementary material, Data S1). We have chosen this matrix because it is an up-to-date version of the dataset from Xu *et al.* [64] and originated from the series of datasets produced by Carballido and colleagues, including many neosauropod taxa such as some Middle Jurassic diplodocoid *Lingwulong* from China, thus giving *Yuzhoulong qurenensis* wide freedom to cluster anywhere within known sauropod diversity. Additionally, we have chosen three other data matrixes from Carballido *et al.* [77], Mannion *et al.* [78] and GEA (originated from González-Riga *et al.* [79]) from Moore *et al.* [80] (electronic supplementary material, Data S2–S4). The data matrix of Carballido *et al.* [77] is the most up-to-date version of Carballido *et al.* which samples a phylogenetically and a spatio-temporally wide array of sauropodomorph taxa, and it will give our specimen yield insights for the placement. The data matrixes from Mannion *et al.* [78] and GEA of Moore *et al.* [80] are some of the largest available for eusauropods. These two datasets are a sampling of neosauropod emphasized and sampling of mamenchisaurid emphasized, respectively. These provide a suitable test for our hypothesis that the taxon represents a neosauropod macronarian, rather than a eusauropod mamenchisaurid.

**Figure 8.**
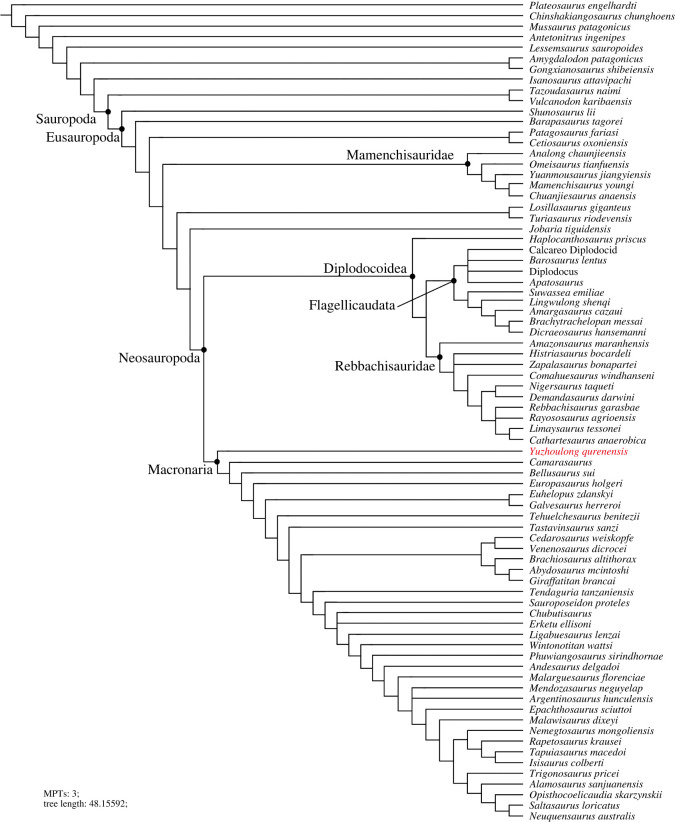
Strict consensus tree of EIW analysis from the main data matrix.

We employed the analyses using equal weights parsimony (EWP) and extended implied weighting (EIW) analyses (we use the concavity constant (K) of 12 referred to Moore *et al.* [80]) for each dataset [80–83]. The EWP analysis of the main data matrix produced 6 most parsimonious trees (MPTs) with a length of 1224 steps ((consistency index (CI) = 0.373; retention index (RI) = 0.702) with a generally good resolution of the tree that supports *Yuzhoulong qurenensis* recovered within Neosauropoda, as a member of Macronaria (figure 7). The Neosauropoda clade is supported by four unambiguous synapomorphies (‘0’ to ‘1’ for characters 96, 120 and 225; ‘0’ to ‘2’ for character 106). The Macronaria clade is supported by two unambiguous synapomorphies (‘0’ to ‘1’ for characters 162, 288). *Yuzhoulong qurenensis* shares all three characters: ‘middle and posterior dorsal centrum in the transverse section are slightly dorsoventrally compressed (character 162)’; ‘length of puboischial is about the one-half total length of pubis (character 288)’. The EIW analysis of the main data matrix produced 3 MPTs with a length of 48.15592 steps (CI = 0.373; RI = 0.702) and a well-resolved strict consensus (figure 8). In our EIW analysis, *Yuzhoulong qurenensis* is located at the basal-most position of the Macronaria clade, supported by two unambiguous characters (similar to the characters of that in EWP analysis).

**Figure. 9.**
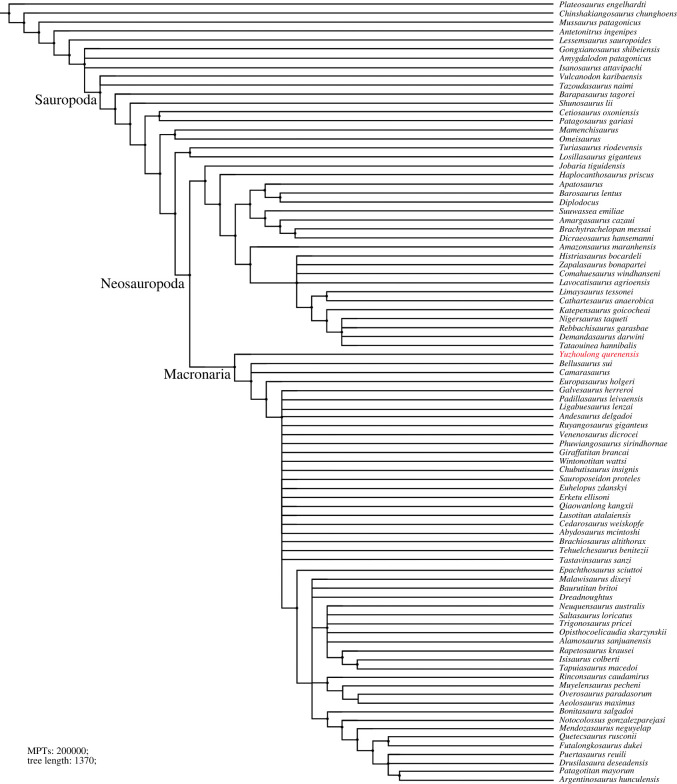
Strict consensus tree of EWP analysis from supplementary data matrix (Carballido *et al.* [77]).

**Figure 10.**
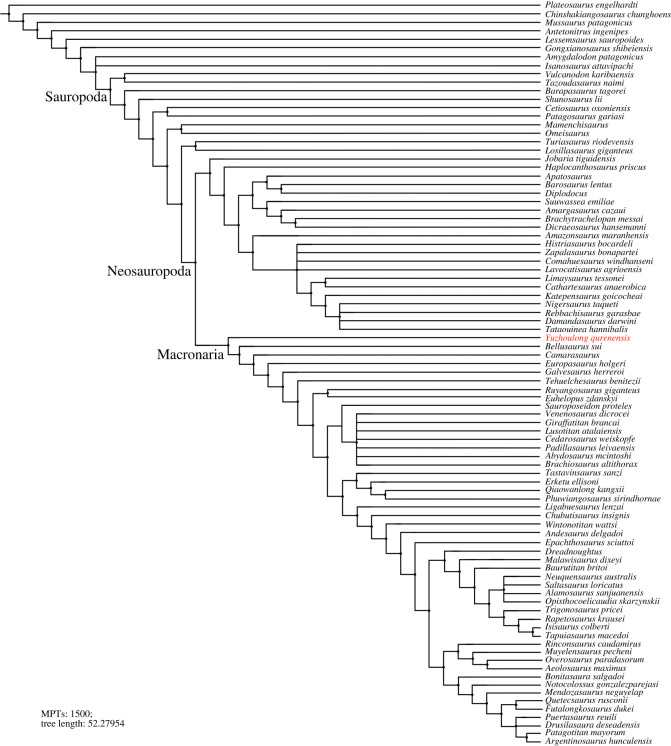
Strict consensus tree of EIW analysis from supplementary data matrix (Carballido *et al.* [77]).

Furthermore, EWP and EIW analyses ran in the referred datasets of Carballido *et al.* [77], Mannion *et al.* [78] and GEA of Moore *et al.* [80]. The EWP analysis of Carballido *et al.* [77] produced 200 000 MPTs with tree lengths of 1370 steps (figure 9). The strict consensus of the result is limited resolution in some parts of the tree, but *Yuzhoulong qurenensis* was recovered within Neosauropoda, as a member of Macronaria with well-resolved in this part. The Neosauropoda clade is supported by four unambiguous synapomorphies (‘0’ to ‘1’ for character 136, 269; ‘0’ to ‘2’ for character 115; ‘1’ to ‘0’ for character 194). The Macronaria clade is supported by five unambiguous synapomorphies (‘0’ to ‘1’ for characters 195, 237, 337, 342; ‘1’ to ‘0’ for character 174). *Yuzhoulong qurenensis* shares all three characters: ‘the minimum width of anterior dorsal neural spine/the length of the anterior dorsal neural spine is 0.5 or greater (present stout and short neural spine (character 174)’; ‘middle and posterior dorsal centra in the transverse section are slightly dorsoventrally compressed (height/width ratio between 0.8 and 1.0) (character 195)’; ‘transverse breadth of anterior caudal neural spines are greater than anteroposterior length (character 237)’; ‘puboischial contact is one half total length of pubis (character 337)’; ‘Ischia pubic articulation greater than the anteroposterior length of the pubic pedicel (character 342)’. The EIW analysis of Carballido *et al.* [77] produced 1500 MPTs with tree lengths of 52.27954 steps (figure 10). The strict consensus of the result is limited resolution in some parts of the tree, but *Yuzhoulong qurenensis* was recovered within Neosauropoda, as a member of Macronaria well-resolved in this part. The Neosauropoda clade is supported by four unambiguous synapomorphies (‘0’ to ‘1’ for character 136, 269; ‘0’ to ‘2’ for character 115; ‘1’ to ‘0’ for character 194). The Macronaria clade is supported by six unambiguous synapomorphies (‘0’ to ‘1’ for characters 195, 237, 337, 342; ‘1’ to ‘0’ for character 174; ‘2’ to ‘0’ for character 176). *Yuzhoulong qurenensis* shares all six characters, with two new characters compared with the EWP analysis: ‘dorsal edge of the anterior dorsal neural spine is flat (character 176)’; ischia pubic articulation greater than the anteroposterior length of the pubic pedicel (character 342).

**Figure 11.**
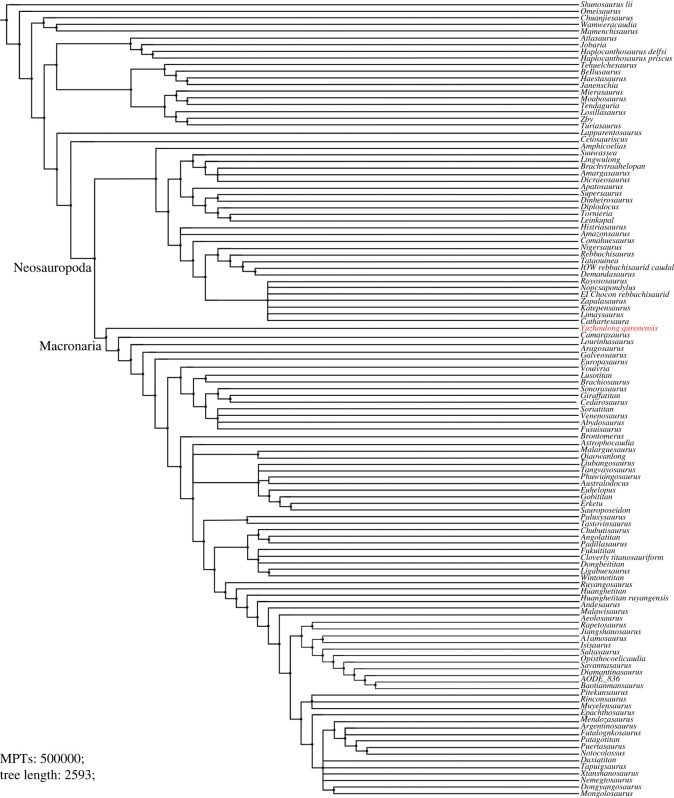
Reduced consensus (50% major consensus) tree of EWP analysis from supplementary data matrix (Mannion *et al.* [78]).

**Figure 12.**
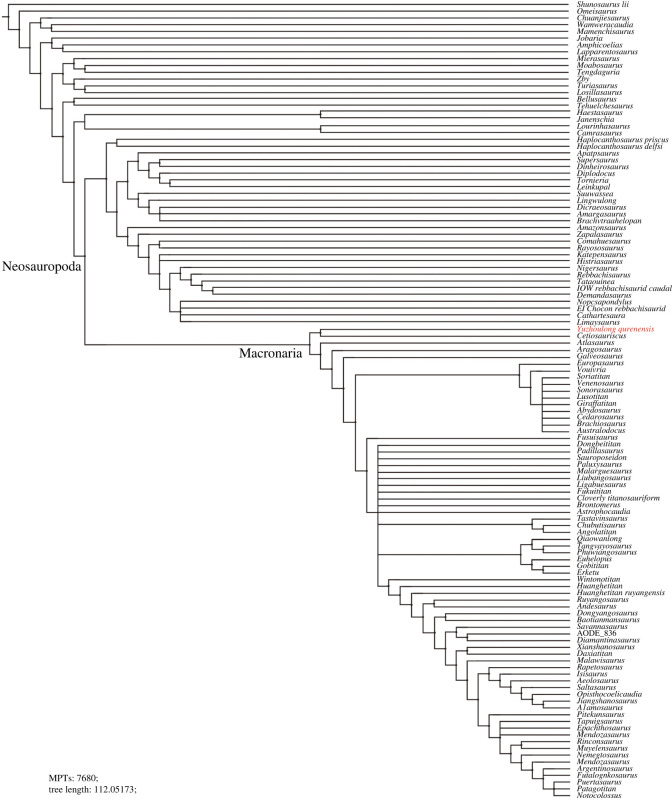
Strict consensus tree of EIW analysis from supplementary data matrix (Mannion *et al.* [78]).

The EWP analysis of Mannion *et al.* [78] produced 500 000 MPTs with the tree lengths of 2593 steps (figure 11). The strict consensus of the result is limited resolution in almost all parts of the tree, then we use the 50% major consensus, but *Yuzhoulong qurenensis* was recovered within Neosauropoda. The Neosauropoda clade is supported by three unambiguous synapomorphies (‘0’ to ‘1’ for character 25, 144, 266). The Macronaria clade is supported by one unambiguous synapomorphy (‘0’ to ‘1’ for characters 335), and *Yuzhoulong qurenensis* is located at the basalmost position. The EIW analysis of Mannion *et al.* [78] produced 7680 MPTs with tree lengths of 112.05173 steps (figure 12). The strict consensus is generally well-resolved which supports *Yuzhoulong qurenensis* recovered within Neosauropoda, as a member of Macronaria (figure 12). The Neosauropoda clade is supported by seven unambiguous synapomorphies (‘0’ to ‘1’ for characters 9, 59, 81, 106, 426; ‘1’ to ‘0’ for character 49, 72). The Macronaria clade is supported by three unambiguous synapomorphies (‘0’ to ‘1’ for characters 248; ‘1’ to ‘0’ for characters 372, 531). *Yuzhoulong qurenensis* shares two of the eight characters: ‘the highest point on the dorsal margin of the ilium occurs anterior to the anterior margin of the base of the pubic process (character 248)’; ventral margin of proximal plate of ischium is flat along its length in lateral view (character 531). Furthermore, *Yuzhoulong qurenensis* share three unambiguous synapomorphies (‘0’ to ‘1’ for character 208; ‘1’ to ‘0’ for character 255; ‘2’ to ‘0’ for character 147) with *Cetiosauriscus* to constitute a clade.

**Figure 13.**
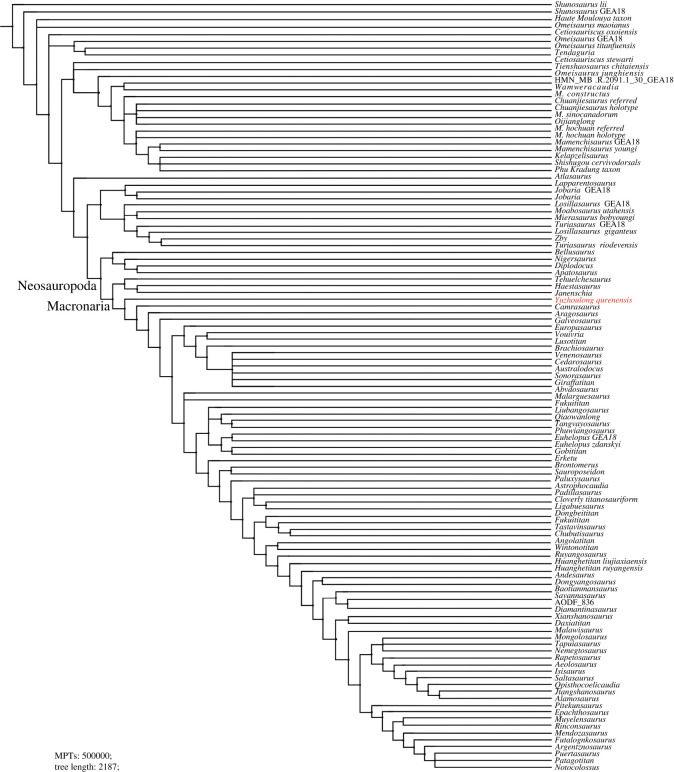
Reduced consensus (50% major consensus) trees of EWP analysis from supplementary data matrix (GEA from Moore *et al.* [80]).

**Figure 14.**
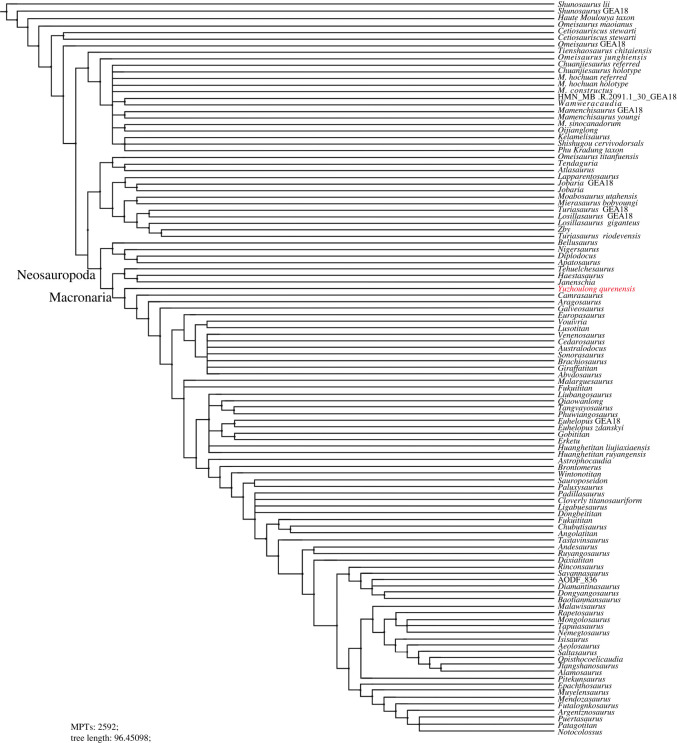
Strict consensus trees of EIW analysis from supplementary data matrix (GEA from Moore *et al.* [80]).

The EWP analysis of GEA from Moore *et al.* [80] produced 5 000 000 MPTs with the tree lengths of 2187 steps (figure 13). The strict consensus of the result is limited resolution in almost all parts of the tree, then we use the 50% major consensus, and *Yuzhoulong qurenensis* was recovered within Neosauropoda, as a member of Macronaria well-resolved in this part. The Neosauropoda clade is supported by nine unambiguous synapomorphies (‘0’ to ‘1’ for characters 16, 52, 133, 266, 285, 341, 366; ‘1’ to ‘0’ for characters 73; ‘1’ to ‘2’ for characters 122). The Macronaria clade is supported by six unambiguous synapomorphies (‘0’ to ‘1’ for characters 155, 171; 267, 335; ‘1’ to ‘0’ for characters 59; 272). *Yuzhoulong qurenensis* shares three of the six characters: dorsoventral height of ischial articulation of the pubis divided by the proximodistal length of the pubis is 0.4 or greater (character 59); middle to posterior dorsal diapophyses are directed laterally or slightly upwards (character 155); Height of anterior neural canal opening of anterior dorsal neural arch is less than the width (character 335). The EWP analysis of GEA from Moore *et al.* [80] produced 2592MPTs with tree lengths of 96.45098 steps (figure 14). The strict consensus of the result is generally well-resolved resolution, and *Yuzhoulong qurenensis* was recovered within Neosauropoda, as the basal-most member of Macronaria well-resolved in this part, similar to the result in EWP analysis.

## 5. Discussion

### 5.1. Relationship of *Yuzhoulong qurenensis* with other relative eusauropods

All of our analyses recovered *Yuzhoulong qurenensis* as a neosauropod, with most placing it in a close relationship with *Camarasaurus* and *Bellusaurus* though details vary (e.g. Figure 7). This position is supported by many unambiguous synapomorphies (e.g. ‘middle and posterior dorsal centrum in the transverse section are slightly dorsoventrally compressed') in the main dataset. It lacks some features that unite other later diverging macronarians, such as median tubercles are absent in posterior cervical to anterior dorsal bifid neural spines; lateral pleurocoels in the lateral surfaces of sacral centra are absent (present in *Camarasaurus*). Moreover, *Yuzhoulong qurenensis* shares several features with some early diverging non-neosauropod and some diplodocoid taxa such as the anterior dorsal centra are opisthocoelous, and the middle to posterior dorsal centra are amphicoelous. This feature exists in some early diverging non-neosauropods (e.g. *Shunosaurus*) and some diplodocoids (e.g. *Lingwulong*). Furthermore, noting that *Yuzhoulong qurenensis* shares several features with diplodocoid *Cetiosauriscus* such as the proximodistal length of the humerus to the femur is 0.7 or less, and such a short ratio is frequently observed in some diplodocoids (in EWP analysis of the dataset of Mannion *et al.* [78]). In general, these may further indicate the ‘basal’ position of this taxon and the possibly mosaic morphological evolution. The partly familiar morphologies between non-neosauropod eusauropod, macronarian, and diplodocoid taxa indicate the early diversity of feeding strategies or locomotion evolved.

### 5.2. Biogeographic origin for neosauropods

Throughout almost the entire Middle Jurassic, the sea level was globally low, most notably during the Late Bajocian-Bathonian [84]. This spurred the development of large epicontinental basins around Pangaea (e.g. southwestern and part of eastern Laurasia (including parts of Europe and North America, and China)) [85]. Then, the sea level gradually rises globally in Callovian [86], an interval of continental rifting and opening of seaways [87–90]. The relationships between some invertebrate (e.g. ammonoid) diversity patterns and sea-level changes have been demonstrated by several works (e.g. [91–93]). During the latest Bajocian to Bathonian, the immigration of some ammonites and ostracods reflects the opening of seaways as well as illustrating the change from a semi-enclosed inland sea to a continental shelf sea in basins of northwest and central Europe [93,94]. Correspondingly, that transgression may trigger the opening of the marine strait and prevents the big terrestrial animals from dispersing. In addition, the global occurrence data of benthic foraminifera statistically revealed the significant differences in the foraminiferal distribution patterns between Laurasia and Gondwanaland in Bathonian, indicating the potential disconnection in that period [95]. The development of the middle Callovian mounds in northern Tethyan is perhaps best linked to a period of minimum sedimentation rates and sufficient accommodation space resulting from a long-term gradual sea-level rise commencing in the Late Bajocian [96]. Notably, in the early Middle Jurassic, regional uplift of structural highs took place in northern Europe, resulting in a change from marine shelf deposition in the Early Jurassic to widespread emergence, erosion and localized deposition [97]. Besides, in some intervals of the upper Bajocian and Bathonian, signs of sedimentation pauses and/or erosion occur in a form of exhumed concretions (hiatus concretions) that were intensively bored and encrusted in conjunctional regions of Europe, and North America [98–100]. Considering the connection between North and South America [101,102] was probably severed in the late Middle Jurassic, that further indicates possible Pangea was still a landmass before Bathonian. The availability of non-marine environments has created a basal frame or is understood to be the main determinant for biological diversification, dispersion, and adaptive radiation [103]. It could be possible for radiation of some neosauropod lineages. In general, it seems logical to assume that the globally low sea level in the early Middle Jurassic boosts for possibly well-developed sauropod radiation (e.g. Neosauropoda) would have occurred in the Northern Gondwana in or before.

**Figure 15.**
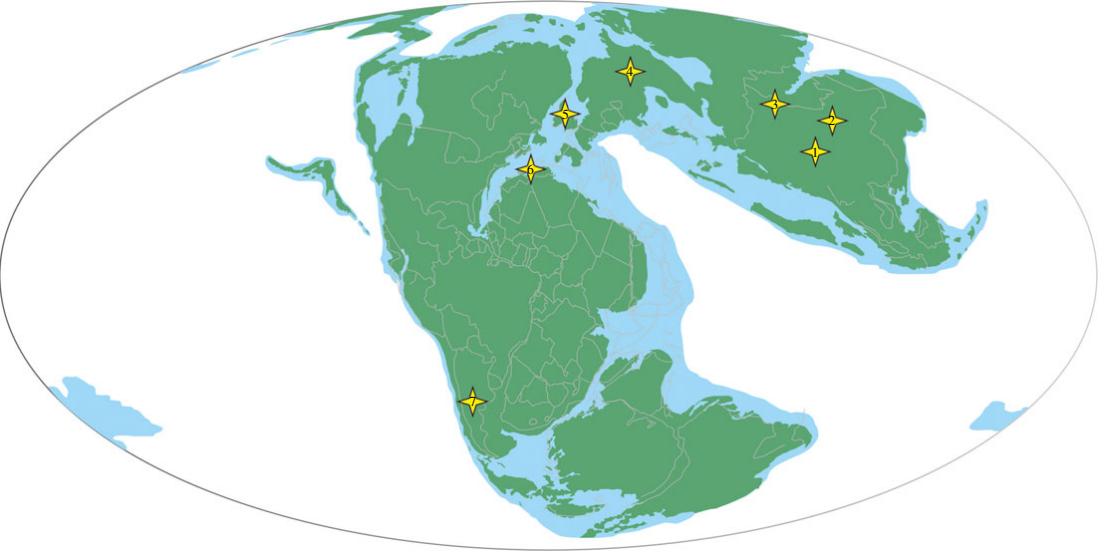
Paleogeographic reconstruction showing the main Middle Jurassic neosauropod records. Paleogeographic reconstruction of 170 Ma from PALEOMAP [116]. Dinosaur faunas are represented with stars. (1), Lower Shaximiao Formation; (2), Zhiluo Formation; (3), Shishugou Formation; (4), Podosinki Formation; (5), Forest Marble Formation; (6), Guettioua Formation; (7), Cañadón Asfalto Formation.

The origin and early diversification of Neosauropoda is one of the most controversial topics in the evolution of Sauropoda [64,78]. In the Middle Jurassic, the non-neosauropod eusauropod taxa are dominating the sauropod faunas globally. The best-known neosauropod taxa are widely distributed in Late Jurassic Laurasia and Gondwana (e.g. diplodocids and some basal macronarians). However, valid Middle Jurassic neosauropods are rarely reported before the discovery of dicraeosaurid *Lingwulong* from middle/late Middle Jurassic (late Bathonian–early Callovian) (The horizon was revised from Yan'an Formation (Aalenian–Bajocian) to Zhiluo Formation (Bathonian–early Oxfordian) [104], then Callovian *Cetiosauriscus stewarti* from Oxford Clay Formation of the United Kingdom [105,106] was phylogenetically recovered as a diplodocid (e.g. [78,107], and this study). Besides them, phylogenetic analyses support the Middle Jurassic African sauropod *Atlasaurus* as a ‘basal’-most member of either Diplodocoidea or macronarian (see also: [80,108,109]), whereas some previous studies have recovered it outside of Neosauropoda [110,111]. *Ferganasaurus*, from the Callovian Balabansai Formation of Kyrgyzstan, was described as a neosauropod [112], but subsequent analysis suggests it was positioned outside the Neosauropoda clade [113]. Two Middle Jurassic Chinese sauropods, *Dashanpusaurus* from the bottom of the Lower Shaximiao Formation, and the other Lower Shaximiao Formation (the specific horizon is unknown) *Bashunosaurus*, were morphologically recovered as macronarians [30,34]. However, these taxa have not been described in detail to include in a phylogenetical analysis, the potential neosauropod position for these taxa still should be treated with caution. Besides these, records of Middle Jurassic neosauropod fragmentary materials were also reported in some regions of Pangea, such as those in the Callovian of the United Kingdom and European Russia [107,114], and Bajocian India [115], that further support neosauropod early diversification and related dispersal events (figure 15). Some putative neosauropod affinities were reported from the late Early Middle Jurassic Patagonia [117] and Middle Jurassic Madagascar [118], perhaps indicating the origin and earliest diversity of Neosauropoda during this period.

In summary, although neosauropods lack global distribution in the Middle Jurassic compared with the prosperous distributions of the Late Jurassic, it may further suggest the timing of its origin and initial diversification could be as early as the late Early Jurassic. The most possible widespread dispersal period is in Bathonian or earlier when the sea level is relatively low. Anyhow, it further undermines the idea of the East Asia Isolation Hypothesis (EAIH).

## 6. Conclusion

The new genus *Yuzhoulong qurenensis* echoes the previous hypothesis, the macronarians readily exist in Middle Jurassic, and this further undermines the EAIH. The morphological comparisons to other macronarians reveal many synapomorphic similarities. Phylogenetical analyses recovered the new taxon as an early diverging macronarian. The discovery of *Yuzhoulong qurenensis* allows for a better understanding of the origin, early evolution and paleogeographic distribution of neosauropods. This study suggests the Middle Jurassic diversity of neosauropods was substantially higher than we previously recognized. and supporting that sauropods achieved a more rapid and varied morphological diversity and palaeogeographical dispersal in the Middle Jurassic.

## Data Availability

The datasets supporting this article have been uploaded to Dryad Digital Repository: https://doi.org/10.5061/dryad.gxd2547ps [119]. The data are provided in electronic supplementary material [120].

## LSID

The electronic version of this article in Portable Document Format (PDF) will represent a published work according to the International Commission on Zoological Nomenclature (ICZN), and hence the new names contained in the electronic version are effectively published under that Code from the electronic edition alone. This published work and the nomenclatural acts it contains have been registered in ZooBank, the online registration system for the ICZN. The ZooBank LSIDs (Life Science Identifiers) can be resolved and the associated information viewed through any standard web browser by appending the LSID to the prefix: http://zoobank.org/. The LSID for this publication is: urn:lsid:zoobank.org:pub:7A790205-07B9-4332-8FBB-9F87D934522E.
